# Assessing Predictive Value of SARS-CoV-2 Epitope-Specific CD8^+^ T-Cell Response in Patients with Severe Symptoms

**DOI:** 10.3390/vaccines12060679

**Published:** 2024-06-18

**Authors:** Cristina Martín-Martín, Estefanía Salgado del Riego, Jose R. Vidal Castiñeira, Maria Soledad Zapico-Gonzalez, Mercedes Rodríguez-Pérez, Viviana Corte-Iglesias, Maria Laura Saiz, Paula Diaz-Bulnes, Dolores Escudero, Beatriz Suárez-Alvarez, Carlos López-Larrea

**Affiliations:** 1 Translational Immunology, Health Research Institute of the Principality of Asturias (ISPA), Avenida de Roma S/N, 33011 Oviedo, Spain; cmartinsorting@finba.es (C.M.-M.); joseramon.vidal@sespa.es (J.R.V.C.); viviana.corte@ispasturias.es (V.C.-I.); marialaura.saiz@ispasturias.es (M.L.S.); paula.bulnes@ispasturias.es (P.D.-B.); 2 Service of Intensive Medicine, Hospital Universitario Central de Asturias, 33011 Oviedo, Spain; estefania.salgado@sespa.es (E.S.d.R.); lolaescudero@telefonica.net (D.E.); 3 Health Research Institute of the Principality of Asturias (ISPA), Avenida de Roma S/N, 33011 Oviedo, Spain; 4 Immunology Department, Hospital Universitario Central de Asturias, 33011 Oviedo, Spain; 5 Microbiology Department, Hospital Universitario Central de Asturias, 33011 Oviedo, Spain; msoledad.zapico@sespa.es (M.S.Z.-G.); mercedes.rodriguezp@sespa.es (M.R.-P.); 6 Translational Microbiology, Health Research Institute of Principado de Asturias (ISPA), 33011 Oviedo, Spain

**Keywords:** SARS-CoV-2, vaccines, pHLA dextramers, epitope-specific CD8^+^ T cell, humoral response, long COVID

## Abstract

Specific T cell responses against SARS-CoV-2 provided an overview of acquired immunity during the pandemic. Anti-SARS-CoV-2 immunity determines the severity of acute illness, but also might be related to the possible persistence of symptoms (long COVID). We retrospectively analyzed ex vivo longitudinal CD8^+^ T cell responses in 26 COVID-19 patients diagnosed with severe disease, initially (1 month) and long-term (10 months), and in a cohort of 32 vaccinated healthcare workers without previous SARS-CoV-2 infection. We used peptide-human leukocyte antigen (pHLA) dextramers recognizing 26 SARS-CoV-2-derived epitopes of viral and other non-structural proteins. Most patients responded to at least one of the peptides studied, mainly derived from non-structural ORF1ab proteins. After 10 months follow-up, CD8^+^ T cell responses were maintained at long term and reaction against certain epitopes (A*01:01-ORF1ab1637) was still detected and functional, showing a memory-like phenotype (CD127+ PD-1+). The total number of SARS-CoV-2-specific CD8^+^ T cells was significantly associated with protection against long COVID in these patients. Compared with vaccination, infected patients showed a less effective immune response to spike protein-derived peptides restricted by HLA. So, the A*01:01-S865 and A*24:02-S1208 dextramers were only recognized in vaccinated individuals. We conclude that initial SARS-CoV-2-specific CD8^+^ T cell response could be used as a marker to understand the evolution of severe disease and post-acute sequelae after SARS-CoV-2 infection.

## 1. Introduction

The clinical outcome of COVID-19 is highly variable, ranging from an asymptomatic or mild disease to a fatal one. This heterogeneity in response depends largely on how the immune system acts during primary infection. For example, an exacerbated systemic immune and inflammatory response that has been described in the most severe and critical forms of the disease may lead to multiple organ dysfunction syndromes [1,2,3]. Patients who develop severe or critical COVID-19 usually present bilateral pneumonia, respiratory failure with low blood oxygenation, and acute respiratory distress syndrome (ARDS) [4]. Recovery from COVID-19 infection is not just hospital discharge or a negative SARS-CoV-2 test result; many post-infection sequelae have been defined [5] and the causes of these symptoms are still undetermined. To define the clinical guidelines for the prevention, diagnosis, follow-up, and rehabilitation of these individuals, we need to gain a better understanding of the underlying mechanisms of long COVID. Moreover, despite the effort since the beginning of the pandemic remains a major challenge to understand how adaptive immune responses develop following infection and vaccination and how they translate into protection against severe forms of the disease.

Early studies suggested that SARS-CoV-2-specific T cells play a key role in COVID-19 disease resolution and in modulating disease severity [6,7]. SARS-CoV-2-specific CD8^+^ T cells had been detected in convalescent patients [8,9,10] and in vaccinated subjects [11,12]. So far as humoral immunity is concerned, SARS-CoV-2-specific antibodies can be found in ∼95% of convalescent COVID-19 patients [13]. However, our knowledge of the adaptive immunity and its contribution to the permanence of post-COVID symptoms is limited.

In this report, we hypothesize that T cell-mediated cytotoxicity might play a role, not only during acute infection, but also in the development of post-acute sequelae of SARS-CoV-2 infection (PASC, also known as “long COVID”). We therefore compare the epitope-specific CD8^+^ T cell responses between a cohort of patients with a severe course of infection and vaccinated healthcare workers without previous COVID-19 infection signs (seronegative before vaccination). Combining different strategies based on multimer pHLA and intracellular cytokine staining (ICS), we thoroughly explore the CD8^+^ T cell-mediated cellular response to COVID-19 and its link with chronic symptomatology.

## 2. Materials and Methods

### 2.1. Study Cohorts

This study includes a cohort of patients (“infection cohort”, n = 26) with a severe course of SARS-CoV-2 infection, recruited from the intensive care unit (ICU) of the Central University Hospital of Asturias (Oviedo, Spain) between March and June 2020. SARS-CoV-2 infection was confirmed after positive PCR testing from oropharyngeal swab and/or SARS-CoV-2 spike IgG-positive antibody testing in the presence of typical symptoms. A severe course of infection was defined as need for respiratory support and consequent transfer to ICU. Initial samples (T = 0) were obtained a median of 21 days (range 8–36 days) after symptom onset, and a second set of samples (T = 10) was collected a median of 10 months after discharge from hospital (range 9–11 months). Post Acute Sequelae of COVID-19 (PASC) was determined for those patients followed by mental health, pneumology, or neurology services, with signs of anxiety, weakness, respiratory distress or altered consciousness among others, persisting for more than 3 months of evolution after hospital discharge. None of the patients manifested these symptoms neither required an appointment to these departments before SARS-CoV-2 infection. 

A second cohort of healthcare workers (HCWs) (“vaccination cohort”, n = 32), with no antecedents of COVID-19 according to previous levels of antibodies against the spike protein and vaccinated with two doses of Comirnaty (BNT162b2), was analyzed. Samples were collected between March and June 2021, at a median of 44 days (range 36–57 days) after the second dose. The demographic and clinical characteristics of both cohorts are summarized in Table 1. The study was carried out in compliance with the regulations of the ethics committee of the Principality of Asturias (CEImPA, n° 2020.521). Signed informed consent was obtained from all participants in compliance with the Helsinki Declaration.

### 2.2. PBMC Isolation and Serum Samples

Venous blood samples were collected from all individuals in appropriate collection tubes. Peripheral blood mononuclear cells (PBMCs) were isolated by density gradient centrifugation (Lymphoprep separation medium, STEMCELL Technologies, Vancouver, Canada) following the manufacturer’s recommendations. Isolated cells were frozen in fetal bovine serum (Gibco, Carlsbad, CA, USA) with 10% DMSO (Sigma-Aldrich, Saint Louis, MO, USA) in liquid nitrogen until used. Serum samples were stored at −80 °C until further analysis. 

### 2.3. HLA Typing

Genomic DNA was isolated from peripheral blood using standard protocols in a Maxwell^®^ Rapid Sample Concentrator (RSC) Instrument (Promega Corp., Madison, WI, USA). HLA typing for HLA-A and HLA-B loci was developed using Lifecodes HLA-SSO typing kits (Immucor Inc., Norcross, GA, USA) based on the Luminex Multi-Analyte Profiling system (xMAP technology) (Luminex Corp., Austin, TX, USA), following the manufacturer’s instructions.

### 2.4. SARS-CoV-2 Epitope Selection and MHC I Dextramer Reagents

Dextramer reagents are based on a dextran polymer backbone covalently bound to multiple MHC-I molecules and the PE fluorochrome. Each one of the MHC-I complexes submit one specific SARS-CoV-2 epitope (Table 2). An exploratory panel of 26 SARS-CoV-2-derived epitopes with the strongest predicted affinity to MHC class I molecules was defined by Immudex (Copenhagen, Denmark). The selection was based on internal predictions using NetMHCpan version 4.0) [14] and on previously published findings, followed by confirmed binding of the selected epitopes to the assigned MHC I molecules for the HLA alleles A*01:01, A*02:01, A*03:01, A*11:01, A*24:02, B*07:02, B*08:01, B*35:01, and B*44:03, which covered most of the alleles in our populations. Additionally, a well-established epitope specific of cytomegalovirus (CMV-pp65 epitope: NLVPMVATV) linked to HLA-A*02:01 was included as a control for compatible patients.

### 2.5. Flow Cytometry Assays

For the pHLA multimer assay and the phenotypic characterization, PBMC samples were thawed and washed with RPMI 1640 (Life Technologies, Carlsbad, CA, USA) supplemented with human serum (Sigma-Aldrich, 10% *v*/*v*), penicillin–streptomycin (Life Technologies, Carlsbad, CA, USA, 1% *v*/*v*) and incubated with LIVE/DEAD Fixable IR Dead Cell Stain Kit (Invitrogen, Waltham, MA, USA) at 37 °C for 30 min. After that, antigen-specific CD8^+^ T cells were stained with pHLA dextramers for 10 min at room temperature. Subsequently, cells were stained with anti-human CD3-PerCP, CD8-FITC, CD127-PeCy7 and PD-1-APC antibodies (all from Biolegend, San Diego, CA, USA) for 20min on ice. Samples were washed twice with PBS before acquisition on a FACS Aria IIu flow cytometer (BD Biosciences, East Rutheford, NJ, USA). All flow cytometric data were analyzed using FlowJo 10.6.0 software (TreeStar, Ashland, OR, USA). 

### 2.6. In Vitro Expansion of SARS-CoV-2-Specific T CD8^+^ Cells

PBMCs were cultured at 37 °C in a 5% CO_2_ atmosphere under stimulation with soluble anti-CD28 antibody (0.5 µg/mL), the corresponding SARS-CoV-2 truncated peptides (10 μg/mL), and recombinant IL-2 (20 UI/mL, Miltenyi Biotech, Cologne, Bergisch Gladbach, Germany). The medium was replaced every 3 days. After 12 days, the cells were re-stimulated with the same peptides (10 μg/mL) in the presence of brefeldin A (0.5 μg/mL, BD GolgiPlug™, BD Biosciences, (East Rutheford, NJ, USA) and CD107a antibody conjugated with PeCy7 (Biolegend) for 4–6 h. A positive control was used, consisting of BD Pharmingen™ Leukocyte activation cocktail, with BD GolgiPlug™ (BD Biosciences), which contains PMA and Ionomycin. Expanded PBMCs were then stained with anti-human CD3-PerCP, CD8-APC and intracellular IFN-γ-FITC antibodies (all from Biolegend) after a fix and perm protocol (Cyto-Fast™ Fix/Perm, Biolegend) according to the manufacturer’s instructions. Samples were acquired in a FACS Aria IIu flow cytometer (BD Biosciences). 

### 2.7. Detection of SARS-CoV-2 Spike-Specific Antibodies

SARS-CoV-2 antibodies were tested against the S protein in sera samples using an automated commercial chemiluminescent system on the LIAISONXL^®^ platform. The LIAISON^®^ SARS-CoV-2 TrimericS IgG assay (DiaSorin, Saluggia, VC, Italy) was used to quantify IgG antibodies to the anti-trimeric spike glycoprotein of SARS-CoV-2 and the analyzer automatically calculates the antibody concentration expressed in arbitrary units per mL (AU/mL), and grades the results as negative (<13 AU/mL) or positive (≥13 AU/mL) with a maximum response of 800 AU/mL. The cPass SARS-CoV-2 Neutralization Antibody Detection Kit (GenScript, Piscataway, NJ, USA) was used to detect neutralizing antibodies, following the manufacturer’s instructions. This test determines the ability of antibodies to block the interaction of the SARS-CoV-2 receptor-binding domain (RBD) and the human ACE2 receptor. Samples were diluted 1:10, run in duplicate, and the percentage inhibition was determined as follow, (1-OD value of sample/OD value of negative control) × 100. A percentage inhibition of <30% was considered to represent an absence of detectable neutralizing antibodies. Moreover, a calibration curve was generated with the SARS-CoV-2 neutralizing antibody calibrator (GenScript) and the semi-quantitative results were shown as units per ml (U/mL). Values ≥ 28.6 U/mL were considered positive.

### 2.8. Statistical Analysis

Statistical analyses were carried out using Prism 9.0 software (GraphPad software, San Diego, CA, USA). The Mann–Whitney or Student’s unpaired samples *t*-test was used to compare differences between groups. Pearson’s correlation coefficients were calculated to examine bivariate associations. Fisher’s exact test was used for contingency analysis of frequency data. Continuous variables are summarized as the mean and standard deviation (SD). Values of *p* < 0.05 were considered to indicate statistical significance.

## 3. Results

### 3.1. Ex Vivo Detection of SARS-CoV-2 Epitope-Specific CD8^+^ T-Cell Response during Acute Infection and Recovery

To evaluate the specific adaptive immune response to COVID-19, we evaluated the SARS-CoV-2-specific CD8^+^ T cell reactivity using technology based on pHLA dextramers. Specifically, we analyzed the presence of specific CD8^+^ T cells against 26 SARS-CoV-2-derived epitopes (Table 2). Patients from the infection cohort were analyzed at different times: an initial time point (T = 0) obtained a median of 21 days after symptom onset, corresponding to the phase of acute infection, and a second one (T = 10) at around 10 months after leaving hospital, corresponding to the convalescent phase. 

**Figure 1:**
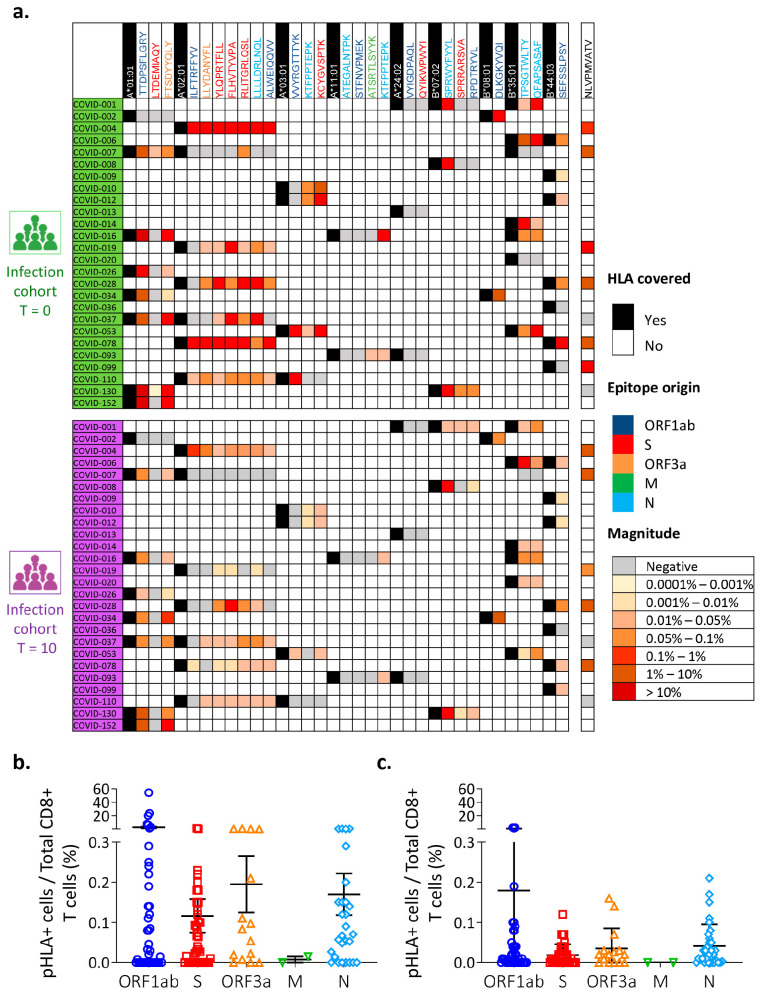
**Ex vivo detection of SARS-CoV2-specific CD8^+^ T response in infected patients at short and long terms.** (**a**) Heatmap of the 129 SARS-CoV-2-specific CD8^+^ T cell responses detected during the acute phase (T = 0) and the recovery phase (T = 10) for infected patients with SARS-CoV-2 (n = 26) using 26 HLA-peptide dextramers. HLA allele-matched responses are represented as the percentage of pHLA+ cells with respect to all CD8^+^ T cells (magnitude, color scale) and the viral protein origin of the peptide (epitope origin, by color). CMV-pp65 response (NLVPMVATV) is included as control in HLA-A02:01 patients. Percentage of pHLA+ cells with respect to the total CD8^+^ T cell number and by origin of viral protein during acute (**b**) and recovery (**c**) phases. Data are summarized as the mean and standard deviation.

In the acute phase, 129 SARS-CoV-2-specific CD8^+^ T cell responses were detected from 26 SARS-CoV-2-derived epitopes. These responses were positive in 84.6% of the patients (22 of the 26) for at least one epitope (Figure 1a). The mean percentage of detected SARS-CoV-2-specific CD8^+^ T cell responses against all epitopes was 0.012% (range: 0–54.4%) of all the CD8^+^ T cells. The specific responses against ORF1ab peptides were those of higher magnitude (2.77%) compared to the specific responses to the ORF3a, spike, membrane, and nucleoprotein, which had mean responses of 0.19%, 0.12%, 0.007%, and 0.17%, respectively (Figure 1b). The specific response mediated by CD8^+^ T cells against the A*01:01/TTDPSFLGRY complex (ORF1ab1637 epitope) was detected in 87.5% (7/8) of the HLA-A*01:01 patients with acute COVID-19 disease (Figure 1a). The magnitude of these CD8^+^ T cells responses was notably high, with an average of 13.9% (range: 0–54.4%) of all the CD8^+^ cells. However, the immune response against other peptides was noticeably lower, with patients showing an average percentage of 0.1–0.5% of specific CD8^+^ T cells against the A*01:01/FTSDYYQLY (ORF3a207), A*02:01/YLQPRTFLL (S269), A*02:01/RLITGRLQSL (S995), A*03:01/KCYGVSPTK (S378), A*11:01/KTFPPTEPK (N361), B*07:02/SPRWYFYYL(N106), B*08:01/DLKGKYVQI(ORF1ab4344), and B*35:01/TPSGTWLTY (N325) complexes. No positive response was found for the A*24:02 allele with the ORF1ab5840 (VYIGDPAQL) and S1208 (QYIKWPWYI) peptides, or with A*11:01/ATEGALNTPK (N134) and A*11:01/STFNVPMEK (ORF1ab2600). Only two patients responded to the A*01:01/LTDEMIAQY (S865) dextramers (Figure 1a).

To investigate whether this specific immune response persists over time, we adopted a similar approach using the samples obtained from these patients during their convalescence phase (T = 10) (Figure 1a). We observed that the magnitude of the long-term immune response was clearly contracted, but that all patients who were initially positive (at T = 0) maintained their specific immune response after 10 months (Figure 1a). The percentage of CD8^+^ T cells specific for each peptide during convalescence was on average about one quarter (0.003%, range: 0–0.6%) of that during the acute phased of the disease (0.012%, range 0–54.4%) (*p* = 0.045) (Figure 1c). Again, the presence of CD8^+^ T cells against the A*01:01/TTDPSFLGRY complex (ORF1ab1637 epitope) was highest (>1% of all CD8^+^ T cells) during convalescence (Figure 1c). 

Therefore, we can conclude that the SARS-CoV-2-specific immune response mediated by CD8^+^ T cells is aimed against peptides derived from the ORF1ab protein, which remains even over the long term, during the convalescence period. 

### 3.2. Functionality of SARS-CoV-2-Specific CD8^+^ T Cells during the Convalescent Phase

It has been postulated that, during a severe SARS-CoV-2 infection, patients experience an immune dysfunction that enables increased viral spread and aggravation of the disease [15]. One of the mechanisms that contributes to the CD8^+^ T cell dysfunction is the overexpression of inhibitory receptors, such as the programmed death-1 (PD-1) molecule, that restrain the effector function, thereby promoting an exhausted state in the T cells. In any case, the functional role of PD-1 expression in CD8^+^ T cells during SARS-CoV-2 acute infection is poorly understood and remains controversial [16]. To characterize the exhaustive phenotype of specific CD8^+^ T cells, we evaluated the combined expression of the PD-1 and the α chain of IL7 receptor (CD127) molecules that allow cells with a memory-like phenotype (CD127+ PD-1+) and terminally exhausted (CD127− PD-1+) T cells to be distinguished [17,18,19].

**Figure 2:**
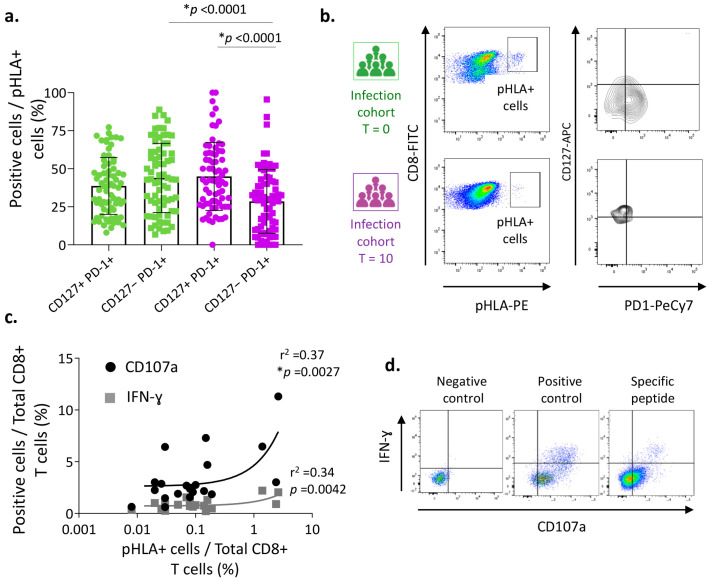
**Phenotype and functionality of SARS-CoV-2-specific CD8^+^ T cells in infected patients.** (**a**) Determination of the percentage of memory-like (CD127+ PD-1+, circles) or terminally exhausted (CD127− PD-1+, squares) cell subsets with respect to the number of pHLA+ T cells detected in peripheral blood samples from infected patients, at short term (T = 0) and long term (T = 10). Of the 129 possible responses studied with respect to the HLA-matched epitope, only the pHLA+ dextramers (n = 86), corresponding to 22 patients, are shown. Data are shown individually and as the mean and standard deviation. (**b**) Representative dot plots showing the gating strategy to identify the different cell subsets based on their CD127 and PD-1 expression, during the acute (T = 0) and recovery (T = 10) phases. (**c**) Correlation between the percentage of cells producing IFN-ɣ (gray squares) and being positive for the CD107a marker (black circles) out of the total number of CD8^+^ T cells, and the percentage of SARS-CoV-2-specific CD8^+^ T cells from the same patient. PBMCs obtained during the recovery phase (T = 10) were treated ex vivo with different specific peptides according to their HLA type. In total, 22 SARS-CoV-2-specific CD8^+^ T cell responses were analyzed. (**d**) Representative dot plots from flow cytometry analysis of PBMCs after ex vivo stimulation with specific peptides (10 µg/mL), without (w/o) stimulation as negative control, or stimulated with BD Pharmingen^TM^ Leukocyte activation cocktail with BD GolgiPlug^TM^ (BD Biosciences, 2 µL) as positive control. Values of *p* < 0.05 were considered significant (*).

We characterized the heterogeneity of the previously determined SARS-CoV-2-specific CD8^+^ T cells in infected patients during the acute (T = 0) and convalescent (T = 10) phases. The percentages of the CD127+ PD-1+ and CD127− PD-1+ cell subsets were nearly similar at T = 0 (Figure 2a,b). However, over the longer term, at T = 10, cells with a memory-like phenotype (CD127+ PD-1+) predominated, being significantly more numerous than exhausted cells (CD127− PD-1+) (*p* < 0.0001). In fact, the CD127− PD-1+ population was significantly smaller during the convalescent phase than in the acute phase (*p* < 0.0001), coinciding with a reduced abundance of terminally exhausted cells (Figure 2a). Therefore, in spite of the contraction of the immune response during convalescence, SARS-CoV-2-specific CD8^+^ T cells display characteristics of a memory-like phenotype. 

To corroborate the functionality of these SARS-CoV-2-specific CD8^+^ T cells during the convalescent phase, we performed functional assays. PBMCs isolated from samples obtained in the recovery phase (T = 10) were activated and expanded with the more immunogenic peptides (A*01-ORF1ab1637, B*08-ORF1ab4344, A*01-ORF3a207, A*02-S269, A*02-S995, A*03-S378, B*07-N106, and B*35-N325), specifically to each patient. After that, the IFN-γ production was evaluated by intracellular cytokine staining (ICS) and the degranulation-related surface marker, CD107a, was also detected (Figure 2c,d). We observed that these specific CD8^+^ T cells detected in the convalescence period were able to respond to further stimulations as implied by the high level of expression of the IFN-γ cytokine and the presence of the CD107a degranulation marker (Figure 2c). Moreover, a significant correlation was observed between the percentage of SARS-CoV-2-specific CD8^+^ T cells detected ex vivo and the number of positive cells producing IFN-γ or stained with anti-CD107a antibody, suggesting the ability of memory-like phenotype cells to respond to new SARS-CoV-2 infections (Figure 2b). 

In conclusion, our results demonstrate that the cellular response mediated by SARS-CoV-2-specific CD8^+^ T cells was lower during the convalescence phase, but maintained a memory phenotype and its functional ability. 

### 3.3. COVID-19 Vaccination Elicited a Higher CD8^+^ T Cell-Mediated Immune Response Than Severe Infection

The question about whether the cellular immune response mediated by SARS-CoV-2-specific CD8^+^ T cells as a consequence of a severe infection is analogous to that obtained after COVID-19 vaccination remained unanswered. To address this, we analyzed the presence of CD8^+^ T cells specific against to the spike protein in a cohort of health care workers (HCWs, n = 32) after they had received two doses of the COVID-19 vaccine. Seven peptides derived from spike protein (Table 2), and according to the HLA typing of each individual, were analyzed. Results were compared with the percentage of spike protein-specific CD8^+^ T cells detected during the convalescence phase (T = 10) in infected patients. This comparison with respect to the HLA-matched epitope proved possible in 20 patients (Figure S1). 

**Figure 3:**
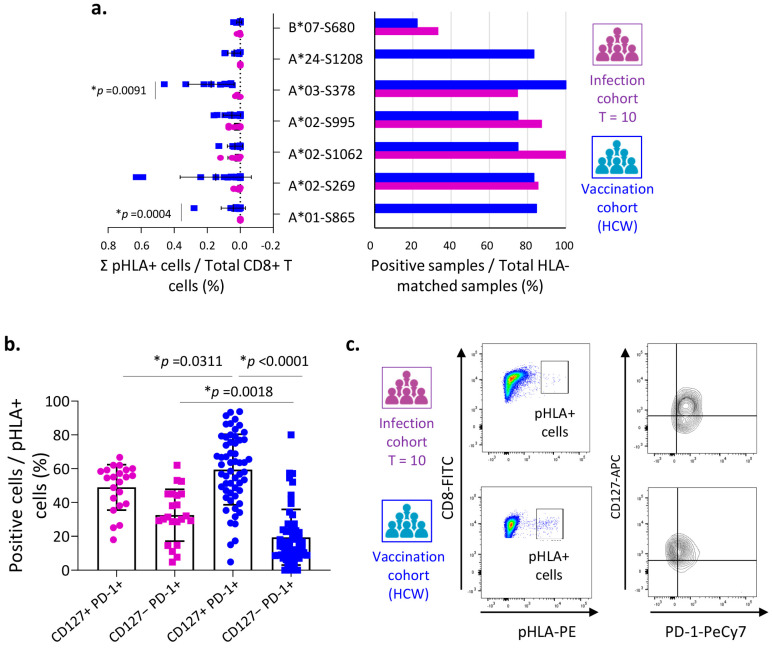
**Spike-specific CD8^+^ T immune response in infection and vaccinated cohorts.** (**a**) Distribution of the positive responses to the HLA-peptide dextramers (pHLA+ cells) specific to the spike protein with respect to the total number of CD8^+^ T cells (left) and number of positive samples for each dextramer out of the total number of HLA-matched analyzed samples (right) in infected patients during the convalescence phase (T =10, purple) and vaccinated healthcare workers (HCWs, blue). (**b**) Determination of the percentage of memory-like (CD127+ PD-1+, circles) or terminally exhausted (CD127− PD-1+. squares) cell subsets with respect to the number of pHLA+ T cells detected against spike protein in peripheral blood samples from infected patients or vaccinated HCWs. Of the 39 possible responses studied according to the HLA-matched epitope in the infected cohort, only the positive pHLA-peptide dextramers (n = 22), corresponding to 10 patients, are shown. For the vaccination cohort, of the 76 possible responses, 55 were positive for spike-specific pHLA-peptide dextramers from 29 individuals. (**c**) Representative dot plots showing the gating strategy used to identify the cell subsets according to the CD127 and PD-1 expression in infected patients (T = 10, purple) and vaccinated HCWs (blue). Data are shown individually and as the mean and standard deviation. Values of *p* < 0.05 were considered significant (*).

A positive response to at least one peptide of the spike protein was found in 50% (10/20) of the SARS-CoV-2-infected patients, compared with 90.6% (29/32) of the vaccinated individuals (Figure S1). The magnitude of the response to spike protein-specific peptides was similar in the two cohorts (infected vs. vaccinated), except for the A*01-S865 (LTDEMIAQY) and A*03-S378 (KCYGVSPTK) HLA–peptide complexes, for which the response was significantly stronger after vaccination (Figure 3a). No infected patient responded to the A*01-S865 and A*24-S1208 dextramers (Figure 3b). The B*07-S680 (SPRRARSVA) was the least well-recognized HLA–peptide complex in both cohorts. 

When we characterized the phenotype of these cells according to their co-expression of CD127 and PD-1, we found that the percentage of cells in the CD127+ PD-1+ subset was significantly higher after vaccination, showing the presence of a greater number of memory-like phenotype CD8^+^ T cells after vaccination (Figure 3b). By contrast, patients with infection showed a significantly higher percentage of CD127−PD-1+ T cells (terminally exhausted) compared with vaccinated patients. 

Due to the differences in sex distribution between both cohorts (infection and vaccination), and considering previous findings suggesting that females had more robust CD8^+^ T cell activation than males after infection with SARS-CoV-2 [20], we compared the phenotype of specific CD8^+^ T cells between males of both cohorts (Figure S2a). Results showed that the CD127+ PD-1+ phenotype is again higher in the case of vaccination, irrespective of sex. Additionally, we compared the total CD8^+^ T-specific response between men and women in the vaccination cohort, and no differences were observed (Figure S2b).

As expected, without exception, all patients and vaccinated HCWs showed the development of anti-S total IgG and neutralizing antibodies (Figure S3a,b). However, the frequency of these antibodies was significantly higher in the group of vaccinated individuals, mainly because of the total anti-S IgG. Moreover, in both cases a significant correlation was observed between the total anti-S IgG and neutralizing antibodies titers, suggesting that both conditions, natural infection and vaccination, are efficient ways of developing a strong humoral response that is capable of blocking the entry of the virus (Figure S3c,d). 

All these data are consistent with the fact that, although a CD8^+^ T cell-mediated immune response specific to protein S is achieved under both infected and vaccinated conditions. However, after vaccination the memory-like phenotype could be available to mount a more robust response upon new exposures. 

### 3.4. The Initial Cellular Response Provides Evidence of Post-Acute Sequelae to SARS-CoV-2 Infection

PASC has recently been identified as a major public health concern, and there is now intense interest in understanding the cause and effects of this condition. In our infection cohort, 10 of the 26 patients (38.5%) developed PASC. The first consultation where compatible signs were observed was on average 34.6 days (range 13–56) post-discharge and maintained for at least three months thereafter. These symptoms included anxiety, weakness, respiratory distress, or altered consciousness among others. The clinical characteristics and severity of initial infection were similar in patients who developed PASC or who recovered fully (Table 3). 

**Figure 4:**
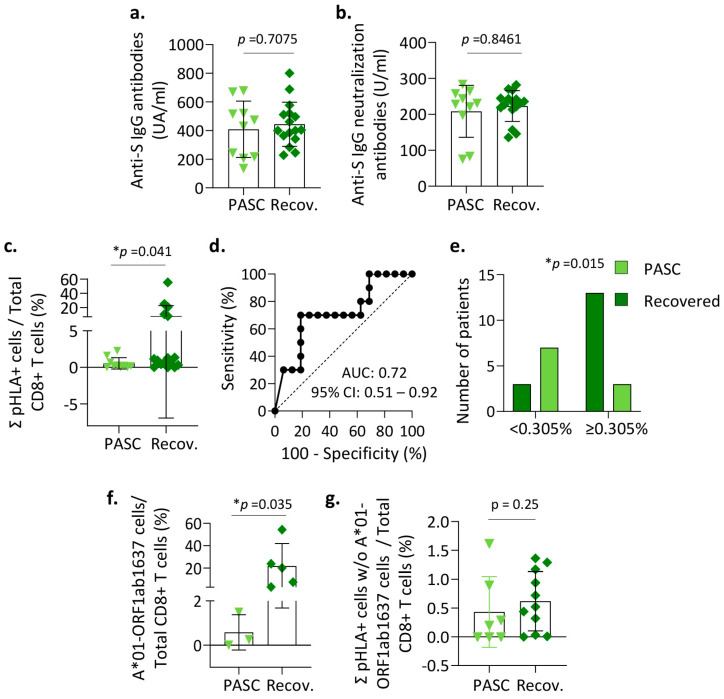
**Humoral and cellular immune response in patients with post-acute sequelae of SARS-CoV-2 infection (PASC).** The total antibody titer against spike protein (**a**) and neutralizing antibodies (**b**) were quantified in serum samples from infected patients at short term (T = 0) who subsequently developed PASC (n = 10, light green triangles), or who did not and fully recovered (n = 16, dark green rhombuses). Red dotted lines indicate the limit of positivity. Data are shown individually and as the mean and standard deviation. (**c**) Distribution of the positive responses against the total of HLA-peptide dextramers (pHLA+ cells) with respect to the total number of CD8^+^ T cells and triggered initially (T = 0) in infected patients who subsequently experienced PASC or who recovered. ROC curve (**d**) and corresponding contingency analysis (**e**), considering the percentage of pHLA+ cells out of total CD8^+^ T cells. Distribution of the positive responses against the specific A*01-ORF1ab1637 dextramer (**f**) or the sum of the rest of dextramers without (w/o) the A*01-ORF1ab1637 CD8^+^ T cells (**g**) and respect to the total number of CD8^+^ T cells and triggered initially (T = 0) in infected patients who subsequently experienced PASC or who recovered. Values of *p* < 0.05 were considered significant (*).

Furthermore, we analyzed whether the initially triggered humoral response or the response mediated by CD8^+^ T cells could be associated with the long-term outcome of these patients. No differences were observed in the titer of total anti-S IgG or neutralizing antibodies developed between patients with good evolution or those who experienced PASC (Figure 4a,b). However, when we analyzed the specific immune response mediated by CD8^+^ T cells, previously determined with the HLA-peptide dextramers, we observed that the percentage of SARS-CoV-2-specific CD8^+^ T cells was lower in patients with PASC compared with those that did not develop PASC (Figure 4c). To analyze in depth whether the initial cellular response mediated by specific CD8^+^ T cells might indicate better or worse disease progression, we performed an ROC curve analysis, obtaining an AUC of 0.72 (Figure 4d). After declaring a cut-off point of 0.305% of the total SARS-CoV-2-specific CD8^+^ T cells, contingency analysis was performed (Figure 4e). It allowed us significantly to differentiate patients who did not develop PASC with a specificity of 70% and a sensitivity of 81%, with an odds ratio of 10. Therefore, and bearing in mind that these results are preliminary, we hypothesize that the cellular immune response mediated by CD8^+^ T cells and triggered initially in response to infection might help predict whether these patients will evolve well or may develop complications over the longer term.

Additionally, we observed that a group of patients (n = 5) with the highest percentage of SARS-CoV-2-specific CD8^+^ T cells reacted with the A*01-ORF1ab1637 (TTDPSFLGRY) dextramer (Figure 4f). By contrast, HLA-matched patients who developed PASC were negative or responded only weakly against this peptide. When eliminating these patients who reacted to this immunodominant peptide, a difference was observed although statistical significance was not reached (Figure 4g), highlighting the relevance of the A*01-ORF1ab1637 CD8^+^ T cells in the cellular response.

## 4. Discussion

There is a growing body of evidence of the protective role of CD8^+^ T cells in SARS-CoV-2 immunity and COVID-19 pathogenesis [21,22,23], but information about their role in PASC is scant or controversial [24,25]. Here, we use pHLA dextramers to analyze the antigen-specific CD8^+^ T cells in patients with severe COVID-19 during the acute and convalescent phases, and to determine their phenotype and functionality after in vitro stimulation using flow cytometry and ICS [26,27]. We employed 26 pHLA dextramers, by covering nine common HLA alleles, that is more than 95% of our population. These epitopes were derived from the ORF1ab, spike, membrane protein, nucleoprotein, and the ORF3a protein. Among the derivatives of ORF1ab, one epitope (TTDPSFLGRY) was included, which displayed strong immunodominant characteristics and whose magnitude of response was substantially greater than the other SARS-CoV-2-specific CD8 T cell responses, as has been previously described [28,29]. To our knowledge, this is the first time that quantification of the number of specific A*01-ORF1ab1637 CD8^+^ T cells, generated early after infection, could be associated with adverse effects (PASC) over the long term. This allows us to generate a hypothesis that should be confirmed in further analysis with a larger cohort. 

Previous studies had emphasized that protein S was not the most immunogenic protein [30,31]. This is an important aspect that needs to be considered because SARS-CoV2 variants and subvariants are continuously emerging and posing a great challenge to current COVID-19 vaccine strategies [32]. For this reason, future vaccine strategies should cover more conserved immunodominant T cell epitopes outside the protein S, such as those we have considered in this study. 

We also demonstrated that not all epitopes derived from the S protein were recognized in the same way in the infected cohort compared with the vaccinated individuals. Furthermore, by phenotypically characterizing these specific cells we have found that both groups of individuals express the PD-1 antigen, which suggests that it is more of an effector marker than an exhausted cell marker, as previously proposed by Rha et al. [16,33]. However, by using the strategy of combining it with the IL7 receptor (CD127), we were able to differentiate those cells, which are known as memory-precursor effector cells during viral infection (PD-1+ CD127+) [17]. In individuals with severe disease and at long term, that cell subset was significantly lower than in vaccinated individuals. We hypothesized that differences in the cellular responses observed in SARS-CoV-2 infection compared with vaccination could be related to the anatomical sites where the viral antigens are encountered, differences in the cell populations stimulated in secondary lymphoid tissues, and potential damage to immunological tissues during infection. When comparing the humoral response, we also observed that the anti-spike IgG titer was higher in vaccinated individuals [34,35]. Nevertheless, we found no link in our study between the level of humoral response and long COVID. 

A major hypothesis generated from this study is that a low level of epitope-specific T CD8^+^ cells in the acute phase of infection may predict the occurrence of long COVID at long term. This observation is in line with the lower frequency of T CD8^+^ cells expressing CD107a in response to nucleocapsid in long COVID, as reported by Peluso et al. [36], and the low level of T CD8^+^ perforin+ reported by Kundura et al. [37]. This may result in defective cytotoxic activity, favoring the replication and persistence of SARS-CoV-2 and, thereby, tissue damage, which could be a potential cause of long COVID. 

We are aware that our study has several limitations, mainly due to the small sample size and the high variability between individuals, so a larger cohort could help to overcome these inconveniences. Nevertheless, further analysis of larger cohorts of long-COVID (PASC) patients are required to test our hypothesis. Specific immunity to the A*01-ORF1ab1637 dextramer is of great interest for discriminating patients with PASC. However, due to the low frequency of HLA-A*01 patients (n = 8) in our cohort, no further analysis is possible. Despite these obstacles, our data clearly demonstrate that patients with severe disease developed an immune response, mediated by SARS-CoV-2-specific CD8 T cells. These cells initially had a more exhausted phenotype, but we showed that longer term, these cells are converted to a memory phenotype and are fully responsive to further restimulations. In summary, to determine the SARS-CoV-2-specific CD8^+^ T cell responses early after severe disease, using pHLA-dextramers could be useful for understanding the evolution of the disease. 

## Figures and Tables

**Table 1:** Demographic and clinical characteristics of the assayed cohorts.

	Infection Cohort	Vaccination Cohort
N° of individuals; n	26	32
Male/Female; n	25/1	8/24
Age; mean (range); y	67.1 (56–78)	40.4 (26–66)
SARS-CoV-2 infection by RT-PCR, n (%)	26 (100%)	0
First sample collection; mean (range); d	21.6 (8–36)	43.5 (36–57)
Comorbidities, n (%)		
Hypertension	14 (53.8%)	0
Diabetes mellitus	4 (15.4%)	1 (3.1%)
Dyslipidemia	9 (34.6%)	3 (9.4%)
Cancer	0	0
Pulmonary disease	3	0
Treatment, n (%)		
Statins	7/26.9%)	3(9.4%)
ARA II	14 (53.8%)	0
Immunosuppressant	0	0

**Table 2:** List of the 26 SARS-CoV-2-specific MHC-I multimers analyzed.

HLA Restriction	Sequence	Protein	Epitope Abbreviation
A*01:01	TTDPSFLGRY	ORF1ab	ORF1ab1637
	LTDEMIAQY	S	S865
	FTSDYYQLY	ORF3a	ORF3a207
A*02:01	ILFTRFFYV	ORF1ab	ORF1ab1637
	LLYDANYFL	ORF3a	ORF3a139
	YLQPRTFLL	S	S269
	FLHVTYVPA	S	S1062
	RLITGRLQSL	S	S995
	LLLLDRLNQL	N	N221
	ALWEIQQVV	ORF1ab	ORF1ab4094
A*03:01	VVYRGTTTYK	ORF1ab	ORF1ab 5538
	KTFPPTEPK	N	N362
	KCYGVSPTK	S	S378
A*11:01	ATEGALNTPK	N	N134
	STFNVPMEK	ORF1ab	ORF1ab2600
	ATSRTLSYYK	M	M171
	KTFPPTEPK	N	N361
A*24:02	VYIGDPAQL	ORF1ab	ORF1ab 5840
	QYIKWPWYI	S	S1208
B*07:02	SPRWYFYYL	N	N105
	SPRRARSVA	S	S680
	RPDTRYVL	ORF1ab	ORF1ab2949
B*08:01	DLKGKYVQI	ORF1ab	ORF1ab4344
B*35:01	TPSGTWLTY	N	N325
	QFAPSASAF	N	N305
B*44:03	SEFSSLPSY	ORF1ab	ORF1ab3946

**Table 3:** Demographic and clinical characteristics of infected patients according to the evolution of the disease.

	PASC	Recovered	*p*
N° of individuals; n	10	16	
Age; mean (range); y	68.6 (58–76)	66.4 (56–78)	0.527
Male / Female; n	10/0	15/1	1
BMI (kg/m^2^); mean (range)	28.9 (24.2–33.8)	29.1 (27.7–33.4)	0.9222
Duration of stay in ICU; d (range)	35.7 (8–85)	22.5 (6–58)	0.1621
Comorbidities, n (%)			
Hypertension	5 (50%)	9 (56.3%)	0.6785
Diabetes mellitus	1 (10%)	3 (18.8%)	1
Dyslipidemia	4 (40%)	5 (31.3%)	0.6924
Cancer	0	0	1
Pulmonary disease	0	3 (18.8%)	0.2615
Laboratory data (admission)			
D-dimer (ng/mL), mean (range)	1752 (1174–2126)	1373 (852.3–6054)	0.8557
Ferritin (ng/mL), mean (range)	1529 (897.8–3792)	1459 (1345–2524)	0.9636
IL6 (pg/mL), mean (range)	136 (74–1487)	77.5 (44.5–302.8)	0.1663
Treatment during ICU stay			
Hydroxychloroquine	6 (60%)	14 (87.5%)	0.1627
Azythromycin	6 (60%)	13 (81.2%)	0.3692
Tocilizumab	6 (60%)	8 (50%)	0.7015
Corticosteroids	6 (60%)	11 (68.8%)	0.6924

n, number; y, years; d, days.

## Data Availability

The manuscript includes all data, presented in tables and figures. Should further data be needed, they can be obtained by contacting with the corresponding author.

## Supplementary Materials

The following supporting information can be downloaded at: https://www.mdpi.com/article/10.3390/vaccines12060679/s1. Figure S1: Ex vivo detection of SARS-CoV2-specific CD8^+^ T response in infected and vaccinated cohorts.; Figure S2: Humoral response in infected and vaccination cohorts.; Figure S3: Humoral response in infected and vaccination cohorts.
