# Supplementary material for: Assessing Predictive Value of SARS-CoV-2 Epitope-Specific CD8+ T-Cell Response in Patients with Severe Symptoms
Source: Vaccines (Basel). 2024 Jun 18;12(6):679. doi: 10.3390/vaccines12060679 (PMC11209605; doi:10.3390/vaccines12060679)
Supplement: Supplementary file 1 [file vaccines-12-00679-s001.zip › vaccines-3034867-supplementary.pdf]

vaccines

MDPI

Article

Assessing Predictive value of SARS-CoV-2 epitope-specific CD8+ T-cell response in patients with severe symptoms

Cristina Martín-Martín [1], Estefanía Salgado del Riego [2,3], Jose R. Vidal Castiñeira [1,4], Maria Soledad Zapico-Gonzalez [5], Mercedes Rodríguez-Pérez [5,6], Viviana Corte-Iglesias [1,4], Maria Laura Saiz [1], Paula Diaz-Bulnes [1], Dolores Escudero [2,6], Beatriz Suárez-Alvarez [1,*], Carlos López-Larrea [1,*].

1 Translational Immunology, Health Research Institute of the Principality of Asturias (ISPA), Avenida de Roma S/N, 33011 Oviedo, Asturias, Spain; cmartinsorting@finba.es (C.M.M.); joseramon.vidal@sespa.es (J.R.V.C.); viviana.corte@ispasturias.es (V.C.-I.); marialaura.saiz@ispasturias.es (M.L.S.); paula.bulnes@ispasturias.es (P.D.-B.)
2 Service of Intensive Medicine, Hospital Universitario Central de Asturias, 33011 Oviedo, Spain; estefania.salgado@sespa.es (E.S.d.R.); lolaescudero@telefonica.net (D.E.)
3 Health Research Institute of the Principality of Asturias (ISPA), Avenida de Roma S/N, 33011 Oviedo, Asturias, Spain
4 Immunology Department, Hospital Universitario Central de Asturias, 33011 Oviedo, Spain
5 Microbiology Department, Hospital Universitario Central de Asturias, 33011 Oviedo, Spain; msoledad.zapico@sespa.es (M.S.Z.-G.); mercedes.rodriguezp@sespa.es (M.R.-P.)
6 Translational Microbiology, Health Research Institute of Principado de Asturias (ISPA), 33011 Oviedo, Spain
* Correspondence: beatriz.suarez@ispasturias.es (B.S.-A.); inmuno@hca.es (C.L.-L.)

.

Citation: To be added by editorial staff during production.

Academic Editor: Firstname Lastname

Received: date
Revised: date
Accepted: date
Published: date

Copyright: © 2024 by the authors. Submitted for possible open access publication under the terms and conditions of the Creative Commons Attribution (CC BY) license (https://creativecommons.org/licenses/by/4.0/).

Supplementary Materials: The following supporting information can be downloaded at: www.mdpi.com/xxx/s1. Figure S1: *Ex vivo* detection of SARS-CoV2-specific CD8+ T response in infected and vaccinated cohorts.; Figure S2: Differences in the spike-specific CD8+ T immune response according to the sex.; Figure S3: Humoral response in infected and vaccination cohorts.

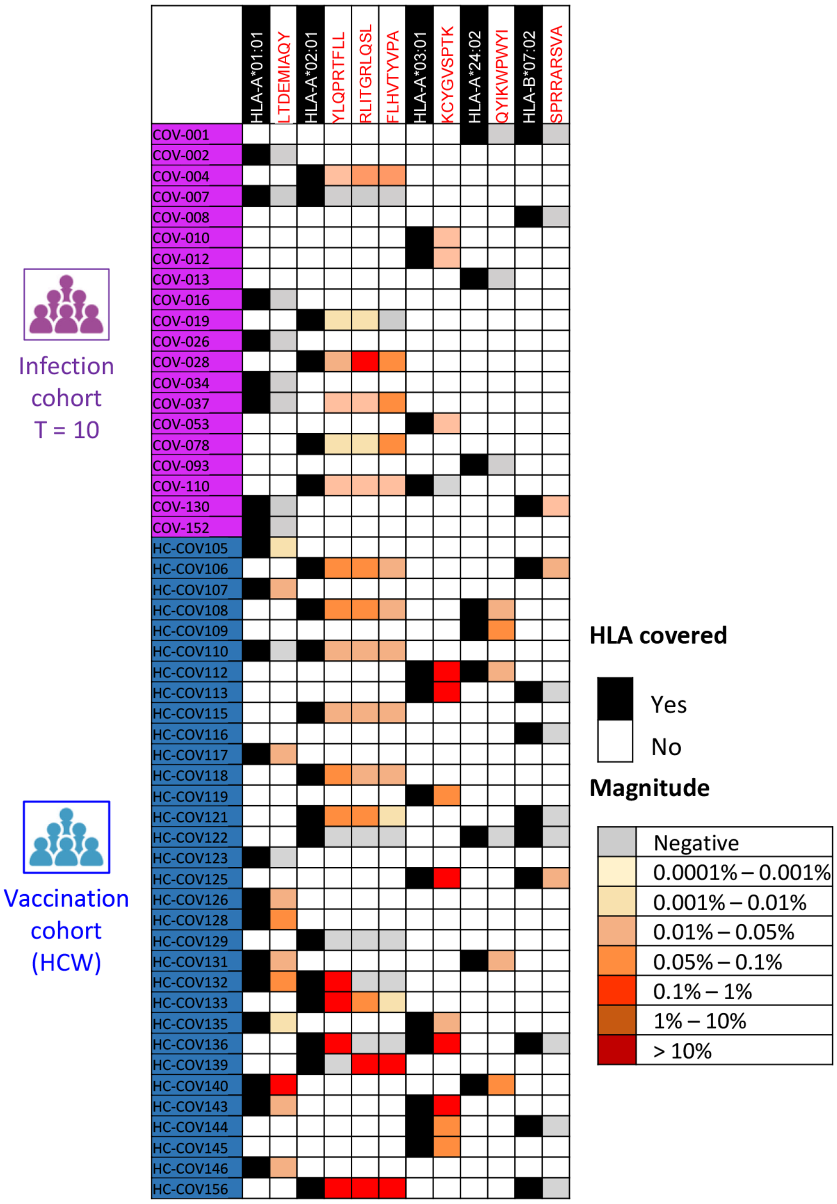

Figure S1 : Ex vivo detection of SARS-CoV2-specific CD8+ T response in infected and vaccinated cohorts. Heatmap of detected spike-specific CD8+ T cell responses detected in infected patients during the convalescent phase (T = 10) and in vaccinated healthcare workers (HCW) after receiving the second dose. For infected patients, a total of 39 possible SARS-CoV-2-specific CD8+ T cell responses were studied corresponding to 20 patients, and 76 responses from 32 individuals for HCW. Positive responses were reached in 10/20 (50%) patients of the infection cohort, and in 29/32 (90.6%) of the HCW. HLA allele-matched responses are presented in relation to their magnitude according to the color scale.

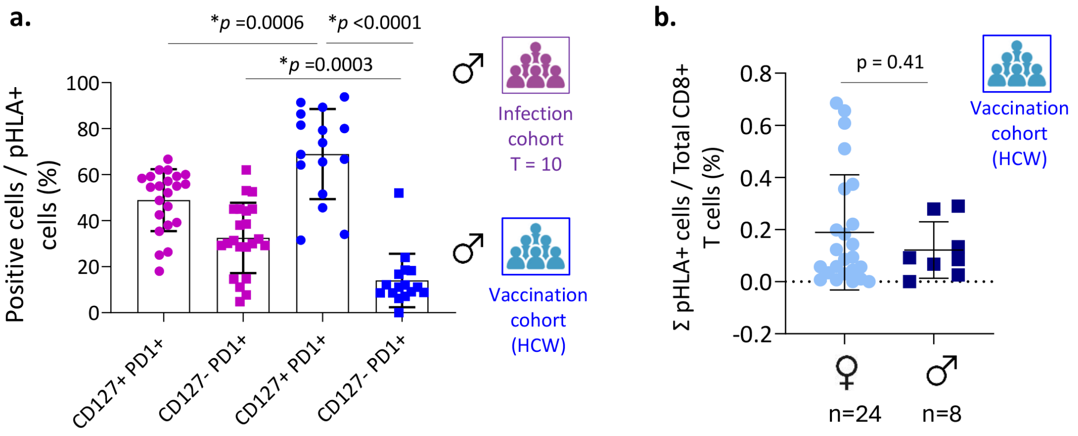

Figure S2: Differences in the spike-specific CD8+ T immune response according to the sex. a. Determination of the percentage of memory-like (CD127+ PD1+) or terminally exhausted (CD127- PD1+) cell subsets with respect to the number of pHLA+ T cells detected against spike protein in peripheral blood samples from males of the infected (n = 25) and vaccinated (n = 8) cohorts. In the infection cohort, among the 25 males, 19 have a HLA molecule linked to S-derived peptides, but only 9 of them showed spike-specific CD8+ T cells, corresponding to 22 positive pHLA-peptide dextramers. From the vaccination cohort, 7 out of 8 males recognized the spike protein (one male was negative for all S-derived peptides analyzed), corresponding to 16 positive spike-specific pHLA-peptide dextramers. **b.** Sum of the pHLA+ cells percentage between females (n = 24) and males (n = 8) in the vaccination cohort. Data are shown individually and as the mean and standard deviation. Values of $p < 0.05$ were considered significant (*).

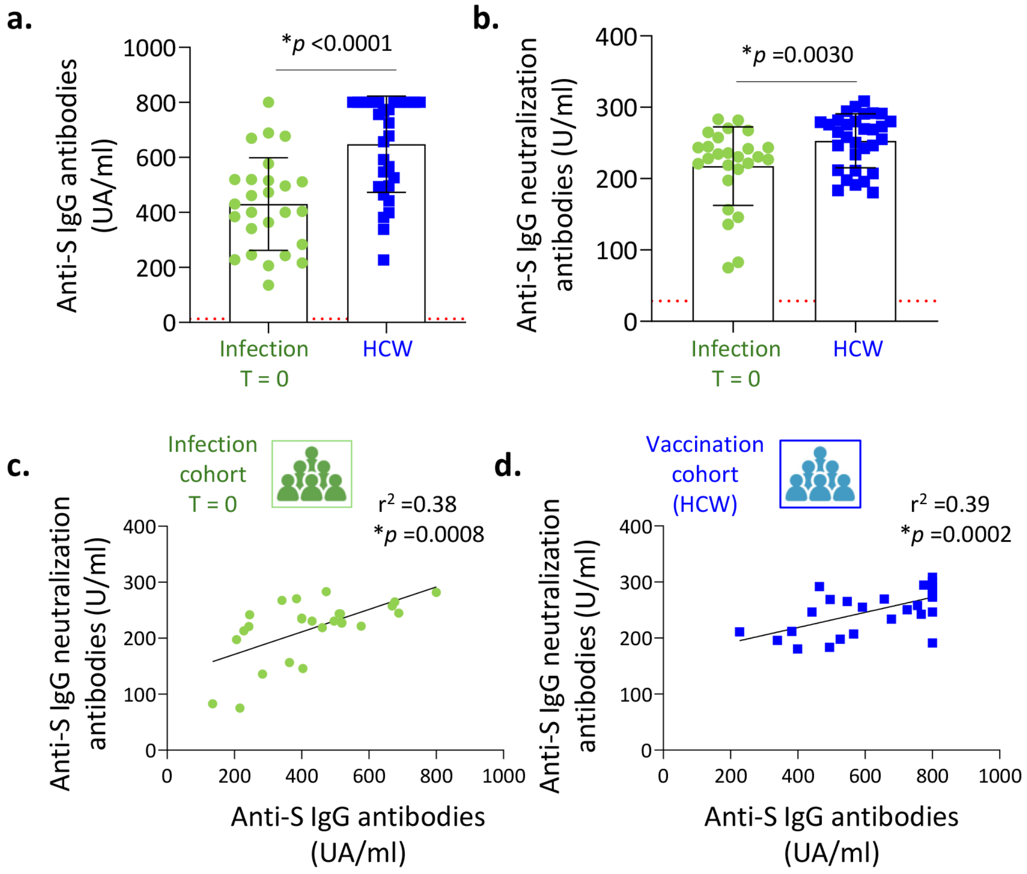

Figure S3: Humoral response in infected and vaccination cohorts. The antibodies total titer against spike protein (**a**) and neutralizing antibodies (**b**) was quantified in serum samples from infected patients at short-times (T=0, n=26, green) and vaccinated HCW (n=32, blue). Red dotted lines mark the limit of positivity. Data are shown individually and as the mean ± standard deviation. Correlation between the frequencies of the titers of total anti-S IgG and neutralizing antibodies in infected (**c**) and vaccination (**d**) cohorts. *P* -values are shown and values $p < 0.05$ were considered significant (*).
